# PROTOCOL: The Impact of Relocation Processes on Populations Facing Socio‐Territorial Inequities: A Scoping Review Protocol: A Systematic Review

**DOI:** 10.1002/cl2.70072

**Published:** 2025-10-13

**Authors:** Pascale Chagnon, Audate Pierre‐Paul, Geneviève Cloutier, Marianne Demers‐Desmarais

**Affiliations:** ^1^ École Supérieur d'aménagement du territoire et de développement régional Université Laval Québec Québec Canada; ^2^ Department of Geography Université de Montréal Montréal Québec Canada; ^3^ Laval University Library Université Laval Québec Québec Canada

**Keywords:** climate adaptation, floods, relocation, scoping review, socio‐territorial inequities

## Abstract

Relocation processes are increasingly considered local adaptations to flooding. While relocation can offer many benefits, it also leads to socio‐economic and socio‐psychological consequences. Moreover, it tends to place greater pressure on populations experiencing socio‐territorial inequities compared to other households. It is therefore important to assess the direct and indirect impacts of relocation to consider its application from a more just and equitable perspective. The objective of this scoping review is to document the impacts of flood‐induced residential relocation processes on populations facing socio‐territorial inequities in North America and Europe. It also seeks to categorize the challenges faced by planners in relocation processes. This will help us better assess the relevance of relocation as an adaptation measure to climate change for populations facing socio‐territorial inequities. This scoping review will be conducted in accordance with the methodological guide *JBI Manual for Evidence Synthesis* (Aromataris and Munn 2020). A systematic search will first be developed in collaboration with an expert librarian. Five databases will be searched: Erudit, Cairn, Web of Science, GreenFILE (EBSCO), and GeoBase (Engineering Village). Gray literature will be collected from Policy Commons, Open Access Theses and Dissertations, Google Scholar, Google, and relevant governmental websites. A screening of the studies and documents obtained will then be carried out by two independent reviewers to retain the relevant documents for the scoping review. If needed, the involvement of a third reviewer will be solicited. The data relevant to our question will then be extracted, using a tool created by the authors, and then analyzed and presented in narrative and tabular formats. The results of this scoping review will be used to discuss and share insights and lessons learned. It will allow us to draw conclusions on the impacts of residential relocation processes on populations facing socio‐territorial inequities and to identify best practices for land‐use planning in the context of climate change.

## 1 Background

Climate change increases extreme weather events, including intense rainfalls that can cause flooding (IPCC 2023). Historically, actions used to face climate risks have often followed the logic: “protect – accommodate – retreat” (Doberstein et al. 2019). Because relocation is often perceived as a form of maladaptation or as politically and socially unpopular, it tends to be the last option considered and implemented (Yarina and Wescoat 2023). In contrast, constructing protective infrastructure in flood‐risk zones remains the primary strategy of decision‐makers and property owners.

In the north‐eastern region of North America, standard mitigation techniques “protect” include sedimentary recharge, ripraps, wave defectors, and the construction of walls or dikes (Friesinger and Bernatchez 2010; Bachand and Comtois 2016; Aerts 2018). Regenerative recharge represents a natural method of counteracting erosion, which contributes to both submersion and flooding. Dikes, in particular, offer a way to preserve buildings and activities from being constantly at risk of being impacted by high tides and floods. However, in many cases, these solutions do not fully address the problem, as structures can break or divert the problem elsewhere. Moreover, they tend to reinforce a false sense of protection. In light of these limitations, retreating and relocation ought to be carefully considered (Biron et al. 2020; Aerts 2018; Menoni and Pesaro 2008).

Residential relocation is defined as a permanent removal of a building from a high‐risk area—affected by floods, in this case—to a low‐risk area. While the process is often initiated by a government decision, it can sometimes come from a citizen's request (Pinter and Rees 2021). Relocation is most often sponsored by a local, regional, or national governmental entity to help households that have experienced flooding. Residents can either relocate to the same municipality or move further away, depending on the options available to them. In any case, relocated households often suffer various types of losses: financial, disruption of their lifestyle, and the weakening of social ties, and so forth (Raikes et al. 2023; Thaler 2021; King et al. 2014).

In theory, residential relocation is strategic in terms of safeguarding values, both for the household and the local or regional administration. From a public health and safety perspective, it can reduce exposure to flood risks and mitigate related mental‐health impacts (Biron et al. 2020; André et al. 2015). Furthermore, it benefits public administrations as it reduces the need for long‐term protection investments and post‐flood reconstruction costs (Boyer‐Villemaire et al. 2021). For many years, researchers have supported relocation as a sustainable planning solution (McGuire and Lynch 2013; King et al. 2014). Decision‐makers are also increasingly considering it in their climate change adaptation planning (O'Donnell 2022).

In practice, however, residential relocation can disrupt individuals' sense of attachment to space and place (Buffin‐Bélanger et al. 2022). In addition, when used as a technical and one‐off solution to reduce risk exposure, relocation tends to exacerbate socio‐territorial inequities and reproduce the very problems it was meant to solve, namely securing individuals and the community (Tubridy et al. 2021). Socio‐territorial inequities are caused by social vulnerability factors, such as age, economic status, belonging to a visible minority, the strength of social ties and networks, as well as from varying levels of exposure to environmental risks. Flood‐risk zones tend to be characterized by high levels of socio‐territorial inequities. As a result, the consequences of flooding tend to be more severe where these inequities are most pronounced (Lynn 2017; Bullard 1990; Bullard and Wright 2018).

When it comes to considering relocation as a response to flood risks, public plans often target materially and socially disadvantaged households, many of which are located in communities that have experienced multiple disasters. This repeated exposure increases their distress and limits their capacity to recover (Dundon and Camp 2021; Lowe et al. 2019; Siders 2019). Furthermore, financial compensations included in relocation programs—when there are any—are not always fair and can disadvantage homeowners (Thaler 2021; Siders 2019). In this context, some studies question the decision‐making processes that lead to relocation plans, while others emphasize that relocation should not be left solely to individual initiative (Guillemot et al. 2014; Rey‐Valette and Rulleau 2016). These concerns raise important issues of social justice, particularly in terms of recognition, procedural, and distributional justice.

Specifically, government relocation programs in North America and Europe seem to disadvantage populations facing socio‐territorial inequities, such as racialized communities, indigenous communities, renters, older people, and so forth. (Thaler 2021). This might be explained by a lack of consideration for social justice issues while planning relocation (Siders 2019; Bullard 1990; Lester et al. 2022). Some studies point to a lack of transparency in the decision‐making processes and reveal that vulnerable populations often feel coerced into accepting relocation programs, regardless of whether or not there are guarantees that buyout conditions will be favorable to the affected households (Siders 2019; Lynn 2017). Since flood‐induced damages are generally more extensive for already precarious or neglected households, relocation programs mostly target lower‐income populations (Siders 2019). In most cases, higher‐income homeowners prefer protection solutions rather than relocation, which is not always financially feasible for poorer households, and even less so for tenants (Lester et al. 2022; Dundon and Camp 2021). Besides, homeowners usually have more social capital and political power to contest relocation decisions (Lester et al. 2022).

The body of knowledge assessing residential relocation as a risk management option remains relatively limited. Existing studies on relocation and its impact on populations often focus on buyout programs (O'Donnell 2022). Although socio‐territorial inequities are increasingly considered by researchers, the ways in which these inequities interact with relocation warrant further study. More precisely, the impacts of planned relocation on living conditions, public health, and the environment should be further assessed. This scoping review seeks to address these gaps and to remedy these shortcomings.

To our knowledge, O'Donnell (2022) is the only systematic review that provides a comprehensive analysis of relocation. Focusing on “managed retreat” and “planned retreat” topics that do not fully encompass the broader scope of relocation. The study highlights that while the relocation topic is increasingly discussed, governance challenges remain, and other risk mitigation methods are still generally preferred. Although social justice dimensions were also addressed in the analysis, they were not the main focus of the systematic review. Building on O'Donnell's (2022) study, our scoping review will focus on the consequences of relocation on populations facing socio‐territorial inequities.

By bringing together existing knowledge of relocation and social justice issues, this review aims to highlight the importance of considering the specific populations targeted by this adaptation measure. Our goal is to emphasize the diverse implications of relocation processes, as this adaptation is increasingly considered by decision‐makers. Indeed, environmental pressures resulting from climate change are compelling us to seriously consider relocation as a means of adaptation, rather than relying on repeated interventions after each flood. The present research will focus on papers that study the situation in North America and Europe to maintain a similar and comparable socio‐political and geographical context.

## 2 Objectives

This scoping review analyzes scientific and gray literature to identify the impacts of residential relocation on affected populations. We are addressing these impacts through the lens of social justice, specifically, recognitional, procedural and distributive justice. This approach aims to place us in a better position to assess the relevance of relocation as a climate change adaptation measure, particularly for populations facing socio‐territorial inequities.

The following research question was developed based on the PCC (Population, Concept, Context) acronym (Aromataris and Munn 2020): What are the impacts of the relocation processes on populations facing socio‐territorial inequities and flood risks?

More specifically, the objectives of this scoping review are to characterize the directs and indirect impacts of relocating at‐risk homes—such as loss of social ties and support networks, stress and mental trauma, financial loss, degradation of territorial integrity and identity, community devitalization, and declining public services—on populations facing socio‐territorial inequities, as well as identifying the related social justice issues.

This scoping review is particularly relevant given the limited amount of research conducted on relocation. Preliminary documentary searches have been conducted in October 2023 and updated in July 2025, in six databases that specialize in systematic reviews or in the field researched (Cochrane Library, Prospero, JBI, GeoBase [Engineering Village], Urban Studies Abstract [EBSCO], Web of Science) to find precedents, if any. These preliminary searches confirmed the importance of a thorough data‐gathering process to analyze issues that warrant further research. Given that the aim of this review is to summarize the current state of knowledge of a still broadly defined topic, the scoping review format was chosen over a systematic review or an evidence gap map.

## 3 Methods

The proposed scoping review will be conducted in accordance with the methodological guide *JBI Manual for Evidence Synthesis* (Aromataris and Munn 2020). The approach will therefore include the following steps:

1. Defining and aligning the objective(s) and question(s).
2. Developing and aligning the inclusion and exclusion criteria with the objective(s) and question(s).
3. Describing the planned approach to evidence searching, selection, data extraction, and presentation of the evidence.
4. Searching for the evidence.
5. Selecting the evidence.
6. Extracting the evidence.
7. Analysis of the evidence.
8. Presentation of the results.
9. Summarizing the evidence in relation to the purpose of the review, making conclusions, and noting any implications of the findings.

Throughout the process, the PRISMA‐ScR checklist will be used to double‐check all necessary elements.

### 3.1 Identification of Relevant Studies

#### 3.1.1 Eligibility Criteria

The papers studied will be found in databases using a keyword‐based search. The words have been extracted from the search question, which was elaborated using the PCC method, as recommended by the *JBI Manual for Evidence Synthesis* (Aromataris and Munn 2020). This framework is used to formulate a clear, relevant, and comprehensive research question that includes all key elements: (1) the population targeted by the study, (2) the concept studied, and (3) the context of the situation. This framework also provides a clear understanding of the eligibility criteria to screen and select articles.

##### 3.1.1.1 Population

The research aims to better understand the reality of populations facing socio‐territorial inequities during residential relocation processes. The studied population consists of households facing socio‐territorial inequities related to factors such as age, gender, economic status, immigrant or ethnic minority background, social network capacity, or residence in at‐risk environments, and so forth. Indeed, this scale is relevant because residential relocation displaces entire households. Moreover, socio‐territorial inequities are often shared among household members. For example, household income that is pooled together to address housing issues, or people making up a household who often face the same type of discrimination (Boelaert et al. 2017). The inclusion criteria, therefore, aim to include articles that address the impacts of relocation on this population level, whether as the primary focus or as part of the sample. In addition, this review will be limited to populations in North America and Europe, given the similar socio‐political contexts of these regions, which allow for meaningful comparisons.

##### 3.1.1.2 Concept

The review's central concept is residential relocation as climate adaptation. As floods increase in number and in intensity with climate change, residential relocation will reveal itself as an adaptation measure of choice from an economic and civil security standpoint. However, this measure does not always follow the precepts of social justice. Therefore, we will seek to include articles that deal with relocation, whether it is the displacement of a residential building, its deconstruction, and the reconstruction of another residential building elsewhere, or its deconstruction and the relocation of the household to another building that already exists. Special consideration will be given to studies examining households that left a residence located in an at‐risk zone and moved to a residence located in another or the same flood‐risk zone. In these cases, adaptation does not take place as the exposure to the risk remains. However, this may be due to social justice‐related issues (e.g., lack of financial means to buy a home that is not in a risk zone or discrimination in rental opportunities), which make them interesting for our study (Dundon and Camp 2021; Siders 2019). Indeed, relocation often affects people from diverse socio‐territorial backgrounds in different ways.

However, this review will exclude articles focusing on out‐of‐country migration. Lastly, it is important to note that our interest lies specifically in the relocation of buildings rather than households. If a household moves, but the building is sold and remains occupied on‐site by others, the intended adaptation has not effectively occurred.

##### 3.1.1.3 Context

We will study relocation within the specific context of land exposed to natural hazards exacerbated by climate change, more particularly, areas exposed to flooding. However, to broaden the research and because there are other hazards whose consequences can be similar to those of floods—either in terms of the temporal aspect of the hazard (linked to climate change, extreme phenomena, rapid events) or in terms of the type of consequences generally experienced (evacuation of one's residence, significant material losses, etc.)—we will also include articles dealing with coastal erosion, hurricanes and landslides. We will exclude articles addressing heat waves, droughts, snow avalanches, forest fires, and tornadoes, nor will we consider articles about natural hazards that are not climate‐related, such as earthquakes and volcanic eruptions, since all these hazards and their consequences on the territory are very different from floods.

#### 3.1.2 Types of Studies

This scoping review will consider all studies published in scientific journals, with the exception of knowledge synthesis articles, including systematic reviews. In these cases, the reviews will be excluded, but their reference lists will be searched to ensure that all relevant articles are included in our review.

Articles without original data will not be included in this scoping review.

The scoping review also seeks to cover gray literature. Government documents or documents produced by recognized non‐governmental organizations will be included. University theses and dissertations, as well as conference proceedings, will also be considered. To this end, the Policy Common database and the government websites of the European Union and the three CUSMA countries will be searched. Documents from the industrial and commercial sectors, such as company balance sheets, will not be considered. Our focus is on the collective and individual impacts of relocation, not on the impacts and consequences for large companies and their assets.

### 3.2 Search Strategy

The search strategy for scientific literature has been developed with the support of a librarian specializing in this method. We used five databases to provide a comprehensive overview of scientific literature on our topic. Hence, Érudit, Cairn, Web of Science Core Collection (SCI‐EXPANDED, SSCI, AHCI, CPCI‐S, CPCI‐SSH, BKCI‐S, BKCI‐SSH, ESCI), GreenFILE (EBSCO), and GeoBase (Engineering Village) will be searched. The last three being self‐explanatory as they are important databases within the field, it is relevant to point out that this scoping review will subsequently be used to complete a PhD project in Québec, Canada, which explains the use of French‐speaking databases (Érudit and Cairn), even if they are more limited. Keywords will be used to search within titles, abstracts, and designated keywords of each article, and the Boolean operator OR will be used to link keywords associated with the same concept. Also, the Boolean operator AND will be used to link concepts. Appendix SI presents an example of the strategy that will be applied to the GeoBase database (Engineering Village). The syntax of the strategy will be modified and adapted to suit each database used. Relevant articles will also be identified through the reference lists of the studies retrieved during the initial searches, and that have passed the selection process described in the following section.

Articles published in languages other than French or English—languages that the authors of this scoping thoroughly understand—will be translated using translation software. An additional concept in the search strategy will enable us to extract only papers dealing with the situation in Europe (European Union plus Great Britain, Switzerland, and Norway) and North America (CUSMA). This strategy is justified by the contextual proximity of the targeted countries, since political structures and the understanding of socio‐territorial inequities may differ in other countries.

Regarding the observed period, we selected 1990 as the first year of publication. It corresponds to the year in which the first report of the Intergovernmental Panel on Climate Change (IPCC) was published, which is a milestone in climate action. For the first time, and following the Brundtland Report, the IPCC brought climate change adaptation issues to the public attention, underlining its social dimension. Therefore, the year 1990 corresponds to a change in the way the issue of adaptation was approached, especially in gray literature.

Regarding the gray literature, Policy Commons, Open Access Theses and Dissertations, as well as Google Scholar and Google, will be used as databases to identify relevant sources. The first two databases will be searched using the same search strategy as the one applied to scientific literature. Additional filters will be applied to limit the selection of publications after 1990, and to the geographical boundaries mentioned above. The same language conditions will be applied.

As far as Google and Google Scholar are concerned, a simplified strategy will be used in unknown mode, as presented in Appendix SII. In these cases, the first 100 search results will be selected for initial filtering.

In addition, further Google searches will be carried out with a filter in the advanced search options to specifically examine the URLs of the targeted countries' governmental websites. The entire research strategy can be found in Appendix SII. The first 50 results of this study will be selected for the first filtering. Only 50 results will be selected here, as our preliminary research indicates that the relevance of the results decreases afterwards.

#### 3.2.1 Study Selection

The selected publications will be transferred to Covidence, a software specifically designed for systematic reviews. A pilot phase of the selection will be carried out by two reviewers who will be assigned the same 20 articles. The results of this pilot phase will be compared before starting the analysis of all articles, to standardize the researchers' approach. If necessary, adjustments will be made to the selection criteria. Any changes will be presented in the scoping review publication.

The initial screening of articles will be carried out by two independent reviewers based on a reading of the paper's titles and abstracts. The inclusion and exclusion criteria presented in the “Eligibility Criteria” section will guide this initial screening. Articles will either be excluded or go on to the next stage, that is, the evaluation of the full text. They will then be included or excluded according to the same criteria. Authors of articles will be contacted if further information on their studies is required. A list of all articles excluded at this point, with reasons for exclusion, will be available at the request of the main author.

Once the first selection of papers has been completed, a second screening process will take place, which involves reading all the papers to select those from which data will be extracted. This step will again begin with a pilot phase, this time involving three articles. The same inclusion and exclusion criteria will be used as in the title and abstract screening, and the same two researchers will conduct this screening process.

Finally, the references cited in the included articles will be scanned to identify relevant publications that may not have been identified by the keyword search. If necessary, the articles identified will go through the same inclusion or exclusion process. The final choice of articles will be discussed between the two reviewers, and any differences of opinion in the selection will be resolved by consensus or with the help of a third party.

Gray literature will simultaneously go through the same process. A PRISMA diagram will then be produced with Covidence, including both scientific and gray literature.

#### 3.2.2 Data Extraction

Once the final selection of articles has been completed, the data extraction phase will begin. The information in Appendix SIII will be identified and collected by two independent researchers. This data extraction instrument might be modified and adapted for each text selected to facilitate the extraction of data presented in different ways. If any adjustments are made, these will be presented in the scoping review. Any differences of opinion between the reviewers will be resolved by consensus or with the help of a third party.

The data extracted will answer the general research question and the objectives of this review. It will include, among other things, specific information on the population (households facing socio‐territorial inequities), the context (buildings in climate hazard risk zones), and the concept (relocation). Relevant findings from the articles will also be extracted.

#### 3.2.3 Data Analysis and Presentation

A PRISMA diagram will be produced to outline the approach and comprehensiveness of the subject's literature. Once the data has been extracted, it will be presented in narrative form in a scientific paper that will outline the characteristics of the selected studies as well as the data extracted and their relationship to our research question and objectives. We will identify the impacts of relocation processes on populations, local management, and the territory, as raised in the selected articles. These results will be compiled in tabular form to facilitate the consideration of these issues in future relocation processes. The results may be presented differently depending on the data collected.

## 4 Results and Discussion

Since the terminologies defining the concept of relocation are varied and change in different contexts, as mentioned by O'Donnell (2022), a possible limitation of this protocol is related to vocabulary. While the search strategy was designed to be as comprehensive as possible, it may have overlooked articles that employed terminology outside our initial scope or unfamiliar to us. Furthermore, although we aim to translate articles written in languages not mastered by the researchers, some relevant studies may still be missed, since we will be using French and English keywords only.

However, we expect that the results of this scoping review will identify gaps in knowledge about the impacts of relocation on populations facing socio‐territorial inequities. The results will guide future research to fill these gaps. Analyses of how socio‐territorial inequities are addressed in the papers will enable us to compile the effects of relocation on different populations and the related social justice issues. We also hope to identify best practices regarding the planning of relocation and adaptation to climate change.

## Author Contributions

This scoping review is carried out as part of the first author's thesis project. She therefore has content expertise, and so does the third author. The second author has, for his part, methodological expertise as he regularly conducts scoping and systematic reviews. The fourth author helped in the development of the search strategy in her capacity as a consulting librarian specializing in bibliographic research. Since our focus is on qualitative data, we do not need statistical expertise.

## Conflicts of Interest

The authors declare no conflicts of interest.

## Sources of Support

This scoping review is carried out as part of the first author's thesis project, which is partly funded by the Réseau Inondation Intersectoriel du Québec (RIISQ) and Mitacs (#IT36789).

## Peer Review

The peer review history for this article is available at https://www.webofscience.com/api/gateway/wos/peer-review/10.1002/cl2.70072.

## Supporting information


**Appendix I:** Search strategy‐scientific literature. **Appendix II:** Search strategy – gray literature. **Appendix III:** Data extraction instrument.

## Data Availability

Data sharing is not applicable to this article as no data sets were generated or analyzed during the current study.

## References

[cl270072-bib-0001] Aerts, J. C. J. H. 2018. “A Review of Cost Estimates for Flood Adaptation.” Water 10, no. 11: 1646. 10.3390/w10111646.

[cl270072-bib-0002] André, C. , P. Sauboua , H. Rey‐Valette , and G. Schauner . 2015. “Acceptabilité Et Mise En Œuvre Des Politiques De Relocalisation Face Aux Risques Littoraux: Perspectives Issues D'une Recherche En Partenariat.” VertigO – la revue électronique en sciences de l'environnement 15, no. 1: 1–22. 10.4000/vertigo.16074.

[cl270072-bib-0003] Aromataris, E. , and Z. Munn . 2020. JBI Manual for Evidence Synthesis. Joanna Briggs Institute. 10.46658/JBIMES-20-01.

[cl270072-bib-0004] Bachand, É. , and S. Comtois . 2016. “Changements Climatiques.” Le Naturaliste Canadien 140, no. 2: 105–112. 10.7202/1036508ar.

[cl270072-bib-0005] Biron, P. , E. Boucher , W. Taha , J.‐L. Martel , and A. Fournier . 2020. Comité expert visant à identifier des solutions porteuses pour la réduction de la vulnérabilité des risques liés à l'inondation par embâcles de glace sur la rivière Chaudière, 103. Ministère de l'Environnement et de la Lutte contre les changements climatiques. https://www.cehq.gouv.qc.ca/zones-inond/rapport/rapport-chaudiere-comite-expert.pdf.

[cl270072-bib-0006] Boelaert, J. , F. Gardes , and S. Langlois . 2017. “Articles.” L'Actualité économique 93, no. 4: 497–529. 10.7202/1058592ar.

[cl270072-bib-0007] Boyer‐Villemaire, U. , A. Lamy , R. Desjardins , et al. 2021. Analyse coûts‐avantages des options d'adaptation aux inondations et aléas fluviaux de la rivière Coaticook à Compton, 168. Ouranos. https://www.ouranos.ca/sites/default/files/2022-07/proj-201419-ge-boyervillemaire-etudecas01.pdf.

[cl270072-bib-0008] Buffin‐Bélanger, T. , D. Maltais , M. Gauthier , et al. 2022. Les inondations au Québec. Presses de l'Université du Québec. https://www.puq.ca/catalogue/livres/les-inondations-quebec-4082.html.

[cl270072-bib-0009] Bullard, R. D. 1990. “Ecological Inequities and the New South: Black Communities Under Siege.” Journal of Ethnic Studies 17, no. 4: 101–115. 11617322

[cl270072-bib-0010] Bullard, R. D. , and B. Wright . 2018. “Race, Place, and the Environment in Post‐Katrina New Orleans.” In Race, Place, and Environmental Justice After Hurricane Katrina, 1st ed., edited by R. D. Bullard and B. Wright , 17–48. Routledge. 10.4324/9780429497858-1.

[cl270072-bib-0011] Doberstein, B. , J. Fitzgibbons , and C. Mitchell . 2019. “Protect, Accommodate, Retreat or Avoid (PARA): Canadian Community Options for Flood Disaster Risk Reduction and Flood Resilience.” Natural Hazards 98, no. 1: 31–50. 10.1007/s11069-018-3529-z.

[cl270072-bib-0012] Dundon, L. A. , and J. S. Camp . 2021. “Climate Justice and Home‐Buyout Programs: Renters as a Forgotten Population in Managed Retreat Actions.” Journal of Environmental Studies and Sciences 11, no. 3: 420–433. 10.1007/s13412-021-00691-4. 33968597 PMC8092994

[cl270072-bib-0013] Friesinger, S. , and P. Bernatchez . 2010. “Perceptions of Gulf of St. Lawrence Coastal Communities Confronting Environmental Change: Hazards and Adaptation, Québec, Canada.” Ocean & Coastal Management 53, no. 11: 669–678. 10.1016/j.ocecoaman.2010.09.001.

[cl270072-bib-0014] Guillemot, J. , E. Mayrand , J. Gillet , and M. Aubé . 2014. “La Perception Du Risque Et L'engagement Dans Des Stratégies D'adaptation Aux Changements Climatiques Dans Deux Communautés Côtières De La Péninsule Acadienne.” VertigO – la revue électronique en sciences de l'environnement 14, no. 2: 1–35. 10.4000/vertigo.15164.

[cl270072-bib-0015] IPCC . 2023. AR6 Synthesis Report: Climate Change 2023. IPCC. https://www.ipcc.ch/report/ar6/syr/.

[cl270072-bib-0016] King, D. , D. Bird , K. Haynes , et al. 2014. “Voluntary Relocation as an Adaptation Strategy to Extreme Weather Events.” International Journal of Disaster Risk Reduction 8: 83–90. 10.1016/j.ijdrr.2014.02.006.

[cl270072-bib-0017] Lester, C. , G. Griggs , K. Patsch , and R. Anderson . 2022. “Shoreline Retreat in California: Taking a Step Back.” Journal of Coastal Research 38, no. 6: 1207–1230.

[cl270072-bib-0018] Lowe, S. R. , J. A. McGrath , M. N. Young , et al. 2019. “Cumulative Disaster Exposure and Mental and Physical Health Symptoms Among a Large Sample of Gulf Coast Residents.” Journal of Traumatic Stress 32, no. 2: 196–205. 10.1002/jts.22392. 30913348 PMC6476642

[cl270072-bib-0019] Lynn, K. 2017. “Rising Recreancy: Flood Control and Community Relocation in Houston, TX, From an Environmental Justice Perspective.” Local Environment 22, no. 3: 321–334. 10.1080/13549839.2016.1195802.

[cl270072-bib-0020] McGuire, C. J. , and D. Lynch . 2013. “Thinking Ahead: The Impacts of Sea Level Rise on Coastal Landscape Protections.” Natural Resources and Environment 27 no. 4: 28–35. https://www.semanticscholar.org/paper/Thinking-Ahead%3A-The-Impacts-of-Sea-Level-Rise-on-McGuire-Lynch/3603b7f013b125f389bd99d2d826e1a6fcb990a9.

[cl270072-bib-0021] Menoni, S. , and G. Pesaro . 2008. “Is Relocation a Good Answer to Prevent Risk?: Criteria to Help Decision Makers Choose Candidates for Relocation in Areas Exposed to High Hydrogeological Hazards.” Disaster Prevention and Management: An International Journal 17, no. 1: 33–53. 10.1108/09653560810855865.

[cl270072-bib-0022] O'Donnell, T. 2022. “Managed Retreat and Planned Retreat: A Systematic Literature Review.” Philosophical Transactions of the Royal Society of London, Series B: Biological Sciences 377, no. 1854: 20210129. 10.1098/rstb.2021.0129. 35574844 PMC9108935

[cl270072-bib-0023] Pinter, N. , and J. C. Rees . 2021. “Assessing Managed Flood Retreat and Community Relocation in the Midwest USA.” Natural Hazards 107, no. 1: 497–518. 10.1007/s11069-021-04592-1.

[cl270072-bib-0024] Raikes, J. , D. Henstra , and J. Thistlethwaite . 2023. “Managed Retreat From High‐Risk Flood Areas: Exploring Public Attitudes and Expectations About Property Buyouts.” Environmental Hazards 22, no. 2: 136–151. 10.1080/17477891.2022.2095970.

[cl270072-bib-0025] Rey‐Valette, H. , and B. Rulleau . 2016. “Gouvernance Des Politiques De Relocalisation Face Au Risque De Montée Du Niveau De La Mer.” Développement durable et territoires. Économie, géographie, politique, droit, sociologie 7, no. 1: 1–16. 10.4000/developpementdurable.11282.

[cl270072-bib-0026] Siders, A. R. 2019. “Social Justice Implications of US Managed Retreat Buyout Programs.” Climatic Change 152, no. 2: 239–257. 10.1007/s10584-018-2272-5.

[cl270072-bib-0027] Thaler, T. 2021. “Just Retreat—How Different Countries Deal With It: Examples From Austria and England.” Journal of Environmental Studies and Sciences 11, no. 3: 412–419. 10.1007/s13412-021-00694-1.

[cl270072-bib-0028] Tubridy, F. , M. Scott , and M. Lennon . 2021. “Managed Retreat in Response to Flooding: Lessons From the Past for Contemporary Climate Change Adaptation.” Planning Perspectives 36, no. 6: 1249–1268.

[cl270072-bib-0029] Yarina, L. , and J. L. Wescoat . 2023. “Spectrums of Relocation: A Typological Framework for Understanding Risk‐Based Relocation Through Space, Time and Power.” Global Environmental Change 79: 102650. 10.1016/j.gloenvcha.2023.102650.

