# Supplementary material for: PROTOCOL: The Impact of Relocation Processes on Populations Facing Socio‐Territorial Inequities: A Scoping Review Protocol: A Systematic Review
Source: Campbell Syst Rev. 2025 Oct 13;21(4):e70072. doi: 10.1002/cl2.70072 (PMC12516908; doi:10.1002/cl2.70072)
Supplement: Supplementary file 1 — Appendix I: Search strategy‐scientific literature. Appendix II: Search strategy – gray literature. Appendix III: Data extraction instrument. [file CL2-21-e70072-s001.docx]

#

# Appendices

### Appendix I: Search Strategy- Scientific Literature

TABLE 1. Search Strategy for GeoBASE (Engineering Village)

| **GéoBase – Controlled Vocabulary** | | | |
| --- | --- | --- | --- |
| **no** | **Search Strategy** | **field** | **rslts** |
| 1 | indigenous population OR social inclusion OR social exclusion OR social impact OR equity OR marginalization OR racial disparity OR gender disparity OR womens status OR low income population OR social justice OR environmental justice OR gender discrimination OR social discrimination OR racial discimination OR age discrimination | Controlled  terms |  |
| 2 | ((social NEAR/3 vulnerabilit*) OR unfairness OR (social* NEAR/3 inequal*) OR (social* NEAR/3 equal*) OR (social* NEAR/3 inequit*) OR (social* NEAR/3 equit*) OR precari* OR (social* NEAR/3 injust*) OR (social* NEAR/3 just*) OR (environmental* NEAR/3 just*) OR (environmental* NEAR/3 injust*) OR (climat* NEAR/3 injust*) OR (climat* NEAR/3 just*) OR underprivileged OR disparity OR discrimin* OR (social* NEAR/3 exclu*) OR (social* NEAR/3 inclu*) OR marginal* OR indigenous OR "first people" OR "first nation*" OR "native people" OR uproot* OR women OR woman OR child* OR gender OR rac*) | Title |  |
| 3 | ((social NEAR/3 vulnerabilit*) OR unfairness OR (social* NEAR/3 inequal*) OR (social* NEAR/3 equal*) OR (social* NEAR/3 inequit*) OR (social* NEAR/3 equit*) OR precari* OR (social* NEAR/3 injust*) OR (social* NEAR/3 just*) OR (environmental* NEAR/3 just*) OR (environmental* NEAR/3 injust*) OR (climat* NEAR/3 injust*) OR (climat* NEAR/3 just*) OR underprivileged OR disparity OR discrimin* OR (social* NEAR/3 exclu*) OR (social* NEAR/3 inclu*) OR marginal* OR indigenous OR "first people" OR "first nation*" OR "native people" OR uproot* OR women OR woman OR child* OR gender OR rac*) | Abstract |  |
| 4 | ((social NEAR/3 vulnerabilit*) OR unfairness OR (social* NEAR/3 inequal*) OR (social* NEAR/3 equal*) OR (social* NEAR/3 inequit*) OR (social* NEAR/3 equit*) OR precari* OR (social* NEAR/3 injust*) OR (social* NEAR/3 just*) OR (environmental* NEAR/3 just*) OR (environmental* NEAR/3 injust*) OR (climat* NEAR/3 injust*) OR (climat* NEAR/3 just*) OR underprivileged OR disparity OR discrimin* OR (social* NEAR/3 exclu*) OR (social* NEAR/3 inclu*) OR marginal* OR indigenous OR "first people" OR "first nation*" OR "native people" OR uproot* OR women OR woman OR child* OR gender OR rac*) | Key-Words |  |
| 5 | ("vulnérab* soci*" OR "soci* vulnérab*" OR "inégal* soci" OR "soci* inégal*" OR "égal* soci*" OR "soci* égal*" OR "inéquit* soci*" OR "soci* inéquit*" OR "équit* soci*" OR "soci* équit*" OR "iniquit* soci*" OR "soci* iniquit*" OR "préca* NEAR/3 soci*" OR "préca* économique*" OR "injustic* soci" OR "justic* soci" OR défavoris* OR disparit* OR discrimin* OR "exclusi* NEAR/3 soci" OR "inclusi* NEAR/3 soci" OR marginal* OR autochton* OR "premier* peuple*" OR "première* nation*" OR deracin*) | Title |  |
| 6 | ("vulnérab* social*" OR "social* vulnérab*" OR "inégal* social" OR "social* inégal*" OR "égal* social*" OR "social* égal*" OR "inéquit* social*" OR "social* inéquit*" OR "équit* social*" OR "social* équit*" OR "iniquit* social*" OR "social* iniquit*" OR "préca* NEAR/3 social*" OR "préca* économique*" OR "injustic* social" OR "justic* social" OR défavoris* OR disparit* OR discrimin* OR "exclusi* NEAR/3 social" OR "inclusi* NEAR/3 social" OR marginal* OR autochton* OR "premier* peuple*" OR "première* nation*" OR deracin*) | Abstract |  |
| 7 | ("vulnérab* social*" OR "social* vulnérab*" OR "inégal* social" OR "social* inégal*" OR "égal* social*" OR "social* égal*" OR "inéquit* social*" OR "social* inéquit*" OR "équit* social*" OR "social* équit*" OR "iniquit* social*" OR "social* iniquit*" OR "préca* NEAR/3 social*" OR "préca* économique*" OR "injustic* social" OR "justic* social" OR défavoris* OR disparit* OR discrimin* OR "exclusi* NEAR/3 social" OR "inclusi* NEAR/3 social" OR marginal* OR autochton* OR "premier* peuple*" OR "première* nation*" OR deracin*) | Key-Words |  |
| 8 | **1 OR 2 OR 3 OR 4 OR 5 OR 6 OR 7** |  |  |
| 9 | (flood OR floods OR flooding OR flash flood OR flood control OR flood damage OR ice breakup OR environmental risk OR disaster OR natural hazard OR environnmental hazard OR catastrophic event OR natural disaster OR extreme event OR hurricane OR storm surge OR sea level change OR coastal erosion OR erosion OR landslide) | Controlled  terms |  |
| 10 | flood* OR inundat* OR surge OR "ice jam*" OR "ice break-up*" OR "ice breakup*" OR (natural NERA/3 risk*) OR (natural NEAR/3 catastrophe*) OR (natural NEAR/3 disaster*) OR (climat* NEAR/3 disaster*) OR (climat* NEAR/3 catastrophe*) OR (coast* NEAR/3 risk*) OR (coast* NEAR/3 hazard*) OR (coast* NEAR/3 erosion*) OR (coast* NEAR/3 retreat*) OR (cliff* NESR/3 retreat*) OR (cliff* NEAR/3 ero*) OR hurricane* OR strom* OR (extrem* NEAR/3 rain*) OR (sea level* ris*) OR (sea level* chang*) OR (wave* overtopp*) OR (storm wave*) OR landslide* OR land-slide* OR landslip* | Title |  |
| 11 | flood* OR inundat* OR surge OR "ice jam*" OR "ice break-up*" OR "ice breakup*" OR (natural NERA/3 risk*) OR (natural NEAR/3 catastrophe*) OR (natural NEAR/3 disaster*) OR (climat* NEAR/3 disaster*) OR (climat* NEAR/3 catastrophe*) OR (coast* NEAR/3 risk*) OR (coast* NEAR/3 hazard*) OR (coast* NEAR/3 erosion*) OR (coast* NEAR/3 retreat*) OR (cliff* NESR/3 retreat*) OR (cliff* NEAR/3 ero*) OR hurricane* OR strom* OR (extrem* NEAR/3 rain*) OR (sea level* ris*) OR (sea level* chang*) OR (wave* overtopp*) OR (storm wave*) OR landslide* OR land-slide* OR landslip* | Abstract |  |
| 12 | flood* OR inundat* OR surge OR "ice jam*" OR "ice break-up*" OR "ice breakup*" OR (natural NERA/3 risk*) OR (natural NEAR/3 catastrophe*) OR (natural NEAR/3 disaster*) OR (climat* NEAR/3 disaster*) OR (climat* NEAR/3 catastrophe*) OR (coast* NEAR/3 risk*) OR (coast* NEAR/3 hazard*) OR (coast* NEAR/3 erosion*) OR (coast* NEAR/3 retreat*) OR (cliff* NESR/3 retreat*) OR (cliff* NEAR/3 ero*) OR hurricane* OR strom* OR (extrem* NEAR/3 rain*) OR (sea level* ris*) OR (sea level* chang*) OR (wave* overtopp*) OR (storm wave*) OR landslide* OR land-slide* OR landslip* | Key-Words |  |
| 13 | ("inond*" OR "embacle*" OR "débacle*" OR "débord*" OR "submer*" OR "risque* naturel*" OR "catastroph* naturelle*" OR "desastre* naturel*" OR "désastre* climatique*" OR "catastroph* climatique*" OR "risque* côti*" OR "aléa* côti*" OR "érosion* coti*" OR "recul* coti*" OR "ouragan*" OR "vague* déferlante*" OR "onde* de tempête" OR "mont* des eaux" OR "mont* de l'eau" OR "élévation des eaux" OR "élévation de l'eau" OR "grande* marée*" OR "glissement* de terrain") | Title |  |
| 14 | ("inond*" OR "embacle*" OR "débacle*" OR "débord*" OR "submer*" OR "risque* naturel*" OR "catastroph* naturelle*" OR "desastre* naturel*" OR "désastre* climatique*" OR "catastroph* climatique*" OR "risque* côti*" OR "aléa* côti*" OR "érosion* coti*" OR "recul* coti*" OR "ouragan*" OR "vague* déferlante*" OR "onde* de tempête" OR "mont* des eaux" OR "mont* de l'eau" OR "élévation des eaux" OR "élévation de l'eau" OR "grande* marée*" OR "glissement* de terrain") | Abstract |  |
| 15 | ("inond*" OR "embacle*" OR "débacle*" OR "débord*" OR "submer*" OR "risque* naturel*" OR "catastroph* naturelle*" OR "desastre* naturel*" OR "désastre* climatique*" OR "catastroph* climatique*" OR "risque* côti*" OR "aléa* côti*" OR "érosion* coti*" OR "recul* coti*" OR "ouragan*" OR "vague* déferlante*" OR "onde* de tempête" OR "mont* des eaux" OR "mont* de l'eau" OR "élévation des eaux" OR "élévation de l'eau" OR "grande* marée*" OR "glissement* de terrain") | Key-Words |  |
| 16 | **9 OR 10 OR 11 OR 12 OR 13 OR 14 OR 15** |  |  |
| 17 | (relocation OR migration OR forced migration OR population migration OR displacement OR resettlement policy OR translocation OR demolition OR buyout) | Controlled  terms |  |
| 18 | (relocat* OR delocat* OR (displac* NEAR/3 population*) OR (displac* NEAR/3 resident*) OR (displac* NEAR/3 propert*) OR (displac* NEAR/3 communit*) OR (displac* NEAR/3 household*) OR resettl* OR expropri* OR buyout OR (population NEAR/3 migra*) OR (resident* NEAR/3 migra*) OR (communit* NEAR/3 migra*) OR (household/ NEAR/3 migra*) OR (human* migra*) OR climigration OR "managed retreat" OR "planned retreat") | Title |  |
| 19 | (relocat* OR delocat* OR (displac* NEAR/3 population*) OR (displac* NEAR/3 resident*) OR (displac* NEAR/3 propert*) OR (displac* NEAR/3 communit*) OR (displac* NEAR/3 household*) OR resettl* OR expropri* OR buyout OR (population NEAR/3 migra*) OR (resident* NEAR/3 migra*) OR (communit* NEAR/3 migra*) OR (household/ NEAR/3 migra*) OR (human* migra*) OR climigration OR "managed retreat" OR "planned retreat") | Abstract |  |
| 20 | (relocat* OR delocat* OR (displac* NEAR/3 population*) OR (displac* NEAR/3 resident*) OR (displac* NEAR/3 propert*) OR (displac* NEAR/3 communit*) OR (displac* NEAR/3 household*) OR resettl* OR expropri* OR buyout OR (population NEAR/3 migra*) OR (resident* NEAR/3 migra*) OR (communit* NEAR/3 migra*) OR (household/ NEAR/3 migra*) OR (human* migra*) OR climigration OR "managed retreat" OR "planned retreat") | Key-Words |  |
| 21 | (relocalis* OR délocalis* OR localisation* OR déménag* OR déplac* OR expropri* OR "migr* NEAR/3 humain*" OR transplant* OR "retrait* planifié*" OR "repli* planifié*" OR "recul* planifié*" OR "rachat" OR "rachet*") | Title |  |
| 22 | (relocalis* OR délocalis* OR localisation* OR déménag* OR déplac* OR expropri* OR "migr* NEAR/3 humain*" OR transplant* OR "retrait* planifié*" OR "repli* planifié*" OR "recul* planifié*" OR "rachat" OR "rachet*") | Abstract |  |
| 23 | (relocalis* OR délocalis* OR localisation* OR déménag* OR déplac* OR expropri* OR "migr* NEAR/3 humain*" OR transplant* OR "retrait* planifié*" OR "repli* planifié*" OR "recul* planifié*" OR "rachat" OR "rachet*") | Key-Words |  |
| 24 | **17 OR 18 OR 19 OR 20 OR 21 OR 22 OR 23** |  |  |
| 25 | Canad* OR Yukon OR "Northwest Territories" OR NWT OR Nunavut OR "British Colombia" OR Alberta OR Saskachewan OR Manitiba OR Ontario OR "New Brunswick" OR "Nova Scotia" OR "Prince Edward Island" OR "New Foundland" OR Newfoundland OR Labrador OR Quebec OR "United-States" OR "United States" OR America* OR USA OR "U.S.A" OR "U.S." OR Mexic* OR Europe* OR Austria* OR Belgi* OR Bulgaria* OR Croatia* OR Cyprus OR Cypriot* OR "Czech Republic" OR Czech* OR Denmark OR Dane* OR Danish OR Estonia* OR Finland OR Finnish OR France OR French OR German* OR Greece OR Greek*OR Hungar* OR Ireland OR Irish OR Ital* OR Latvia* OR Lithuania* OR Luxembourg* OR Malt* OR Netherlands OR Dutch OR Poland OR Polish OR Portug* OR Romania* OR Slovak* OR Slovenia* OR Spain OR Spanish OR Sweden OR Swedish OR "United Kingdom" OR UK OR "U.K." OR "Great Britain" OR England OR English OR Scotland OR Scottish OR Wales OR Welsh OR Scandinavi* OR Norway OR Norwegian* OR Switzerland OR swiss | Title |  |
| 26 | Canad* OR Yukon OR "Northwest Territories" OR NWT OR Nunavut OR "British Colombia" OR Alberta OR Saskachewan OR Manitiba OR Ontario OR "New Brunswick" OR "Nova Scotia" OR "Prince Edward Island" OR "New Foundland" OR Newfoundland OR Labrador OR Quebec OR "United-States" OR "United States" OR America* OR USA OR "U.S.A" OR "U.S." OR Mexic* OR Europe* OR Austria* OR Belgi* OR Bulgaria* OR Croatia* OR Cyprus OR Cypriot* OR "Czech Republic" OR Czech* OR Denmark OR Dane* OR Danish OR Estonia* OR Finland OR Finnish OR France OR French OR German* OR Greece OR Greek*OR Hungar* OR Ireland OR Irish OR Ital* OR Latvia* OR Lithuania* OR Luxembourg* OR Malt* OR Netherlands OR Dutch OR Poland OR Polish OR Portug* OR Romania* OR Slovak* OR Slovenia* OR Spain OR Spanish OR Sweden OR Swedish OR "United Kingdom" OR UK OR "U.K." OR "Great Britain" OR England OR English OR Scotland OR Scottish OR Wales OR Welsh OR Scandinavi* OR Norway OR Norwegian* OR Switzerland OR swiss | Abstract |  |
| 27 | Canad* OR Yukon OR "Northwest Territories" OR NWT OR Nunavut OR "British Colombia" OR Alberta OR Saskachewan OR Manitiba OR Ontario OR "New Brunswick" OR "Nova Scotia" OR "Prince Edward Island" OR "New Foundland" OR Newfoundland OR Labrador OR Quebec OR "United-States" OR "United States" OR America* OR USA OR "U.S.A" OR "U.S." OR Mexic* OR Europe* OR Austria* OR Belgi* OR Bulgaria* OR Croatia* OR Cyprus OR Cypriot* OR "Czech Republic" OR Czech* OR Denmark OR Dane* OR Danish OR Estonia* OR Finland OR Finnish OR France OR French OR German* OR Greece OR Greek*OR Hungar* OR Ireland OR Irish OR Ital* OR Latvia* OR Lithuania* OR Luxembourg* OR Malt* OR Netherlands OR Dutch OR Poland OR Polish OR Portug* OR Romania* OR Slovak* OR Slovenia* OR Spain OR Spanish OR Sweden OR Swedish OR "United Kingdom" OR UK OR "U.K." OR "Great Britain" OR England OR English OR Scotland OR Scottish OR Wales OR Welsh OR Scandinavi* OR Norway OR Norwegian* OR Switzerland OR swiss | Key-Words |  |
| 28 | Canad* OR Yukon OR "Territoires du Nord-Ouest" OR TNO OR Nunavut OR "Colombie-Britannique" OR "Colombie Brritannique" OR Alberta OR Saskachewan OR Manitoba OR Ontario OR "Nouveau Brunswick" OR "Nouveau-Brunswick" OR "Nouvelle Écosse" OR "Nouvelle-Écosse" OR "Ile du Prince Édouard" OR "Ile-du-Prince-Édouard" OR "Terre Neuve" OR "Terre-Neuve" OR Labrador OR Québec OR "États-Unis" OR "É-U." OR "É-U. A. " OR Mexi* OR "Amérique du Nord" OR Europ* OR Allema* OR Autrich* OR Belg* OR Bulgar* OR Chypr* OR Croat* OR Danemark OR Danois* OR Espagn* OR Estonie* OR Finland* OR Franc* OR Grèce OR Grèque*OR Hongr* OR Irland* OR Italie* OR Lettonie* OR Lituanie* OR Luxembourg* OR Malt* OR "Pays-Bas" OR Néerland* OR Pologne OR polonais* OR Portuga* OR "République Tchèque" OR Tchequ* OR Rouma* OR Slovaqu* OR Slovénie* OR Suèd* OR "Royaume-Uni" OR "Grande-Bretagne" OR Angleterre OU Anglais* OR Écoss* OR "Pays de Galle" OU gallois* OR Scandinav* OR Norvèg* OR Suisse* | Title |  |
| 29 | Canad* OR Yukon OR "Territoires du Nord-Ouest" OR TNO OR Nunavut OR "Colombie-Britannique" OR "Colombie Brritannique" OR Alberta OR Saskachewan OR Manitoba OR Ontario OR "Nouveau Brunswick" OR "Nouveau-Brunswick" OR "Nouvelle Écosse" OR "Nouvelle-Écosse" OR "Ile du Prince Édouard" OR "Ile-du-Prince-Édouard" OR "Terre Neuve" OR "Terre-Neuve" OR Labrador OR Québec OR "États-Unis" OR "É-U." OR "É-U. A. " OR Mexi* OR "Amérique du Nord" OR Europ* OR Allema* OR Autrich* OR Belg* OR Bulgar* OR Chypr* OR Croat* OR Danemark OR Danois* OR Espagn* OR Estonie* OR Finland* OR Franc* OR Grèce OR Grèque*OR Hongr* OR Irland* OR Italie* OR Lettonie* OR Lituanie* OR Luxembourg* OR Malt* OR "Pays-Bas" OR Néerland* OR Pologne OR polonais* OR Portuga* OR "République Tchèque" OR Tchequ* OR Rouma* OR Slovaqu* OR Slovénie* OR Suèd* OR "Royaume-Uni" OR "Grande-Bretagne" OR Angleterre OU Anglais* OR Écoss* OR "Pays de Galle" OU gallois* OR Scandinav* OR Norvèg* OR Suisse* | Abstract |  |
| 30 | Canad* OR Yukon OR "Territoires du Nord-Ouest" OR TNO OR Nunavut OR "Colombie-Britannique" OR "Colombie Brritannique" OR Alberta OR Saskachewan OR Manitoba OR Ontario OR "Nouveau Brunswick" OR "Nouveau-Brunswick" OR "Nouvelle Écosse" OR "Nouvelle-Écosse" OR "Ile du Prince Édouard" OR "Ile-du-Prince-Édouard" OR "Terre Neuve" OR "Terre-Neuve" OR Labrador OR Québec OR "États-Unis" OR "É-U." OR "É-U. A. " OR Mexi* OR "Amérique du Nord" OR Europ* OR Allema* OR Autrich* OR Belg* OR Bulgar* OR Chypr* OR Croat* OR Danemark OR Danois* OR Espagn* OR Estonie* OR Finland* OR Franc* OR Grèce OR Grèque*OR Hongr* OR Irland* OR Italie* OR Lettonie* OR Lituanie* OR Luxembourg* OR Malt* OR "Pays-Bas" OR Néerland* OR Pologne OR polonais* OR Portuga* OR "République Tchèque" OR Tchequ* OR Rouma* OR Slovaqu* OR Slovénie* OR Suèd* OR "Royaume-Uni" OR "Grande-Bretagne" OR Angleterre OU Anglais* OR Écoss* OR "Pays de Galle" OU gallois* OR Scandinav* OR Norvèg* OR Suisse* | Key-Words |  |
| 31 | **25 OR 26 OR 27 OR 28 OR 29 OR 30** |  |  |
| 32 | **8 AND 16 AND 24 AND 31** |  |  |
|  | filtre date |  |  |

### Appendix II: Search Strategy – Gray Literature

| **Strategy in english** | |
| --- | --- |
| 1 | ("social vulnerabilit*"\|"social* just*"\|"marginal*"\|"indigeous") |
| 2 | ("flood"\|"natural disaster"\|"hurricane*"\|"sea level* ris*") |
| 3 | ("relocat*"\|"resettl*"\|"managed retreat*"\|"buyout*") |
| **4** | **1 AND 2 AND 3** |
| **Stratégie en francais** | |
| A | ("vulnérab* social*"\|"inégal*social*"\|"égal* social*"\|"justice*sociale*"\|"disparit*"\|"discrimin*"\|"autochton*") |
| B | (inond*"\|submer*\|"risque* naturel*") |
| C | (relocalis*\|"expropri*"\|"déménag*") |
| **D** | **A ET B ET C** |

### Appendix III: Data Extraction Instrument

| **Data Extraction** | | | | |
| --- | --- | --- | --- | --- |
| Made by |  | Date |  | |
| **Study Identification** | | | | |
| Title |  | Writer |  | |
| Country |  | Year of publication |  | |
| Type of publication |  | Complete reference |  | |
| **Study Characteristics** | | | | |
| Type of source | Scientific published | Gray - thesis | Gray - government | Gray - other |
| Study objectives |  |  |  | |
| By whom and for who (gray litt.) |  |  |  | |
| **Relocation** | | | | |
| What event caused the relocation? | New at-risk zones cartography | Flooding event | Multiple flooding events | Other natural catastrophes, specify |
| Whose decision was it? | Chosen by the population | Imposed by the state | Mutual agreement | |
| What level of government was implicated? | No government programs | Municipality | Region | State |
| Were the citizen helped by the government? | No | Yes, specify |  | |
| What type of relocation was it? | Building moved (close) | Deconstruction and reconstruction in the same municipality | Resettlement in another municipality | |
| Other information on the relocation |  |  |  | |
| **Population** | | | | |
| What were the social characteristics of the impacted population before the intervention? |  |  |  | |
| What were the social characteristics of the impacted population after the intervention? |  |  |  | |
| **Impacts of relocation** | | | | |
| How are social aspects considered? | Mentioned in the background but not in the subject of the study | Mentioned in the analysis | At the center of the study | |
| What social justice dimensions are implicated? | Recognition | Procedural | Distributive | |
| How are people affected by those dimensions? |  |  |  | |
| What direct impacts were lived by the population during or after the relocation? |  |  |  | |
| What indirect impacts were lived by the population during or after the relocation? |  |  |  | |
| **Authors’ conclusions** | | | | |
| Did the authors identify a lack of consideration for inequities? Which? |  |  |  | |
| What are the authors’ recommendations? |  |  |  | |
| Other interesting studies referred |  |  |  | |
